# Human Papillomavirus 16 Oncoprotein E7 Stimulates UBF1-Mediated rDNA Gene Transcription, Inhibiting a p53-Independent Activity of p14^ARF^

**DOI:** 10.1371/journal.pone.0096136

**Published:** 2014-05-05

**Authors:** Isabelle Dichamp, Paule Séité, Gérard Agius, Alice Barbarin, Agnès Beby-Defaux

**Affiliations:** 1 Unité de Virologie, Centre Hospitalier Universitaire de Poitiers, Faculté de Médecine et Pharmacie, Poitiers, France; 2 Equipe Emergente 2RCT «Récepteurs, Régulations, Cellules Tumorales», Université de Poitiers, Poitiers, France; The University of North Carolina at Chapel Hill, United States of America

## Abstract

High-risk human papillomavirus oncoproteins E6 and E7 play a major role in HPV-related cancers. One of the main functions of E7 is the degradation of pRb, while E6 promotes the degradation of p53, inactivating the p14^ARF^-p53 pathway. pRb and p14^ARF^ can repress ribosomal DNA (rDNA) transcription in part by targeting the Upstream Binding Factor 1 (UBF1), a key factor in the activation of RNA polymerase I machinery. We showed, through ectopic expression and siRNA silencing of p14^ARF^ and/or E7, that E7 stimulates UBF1-mediated rDNA gene transcription, partly because of increased levels of phosphorylated UBF1, preventing the inhibitory function of p14^ARF^. Unexpectedly, activation of rDNA gene transcription was higher in cells co-expressing p14^ARF^ and E7, compared to cells expressing E7 alone. We did not find a difference in P-UBF1 levels that could explain this data. However, p14^ARF^ expression induced E7 to accumulate into the nucleolus, where rDNA transcription takes place, providing an opportunity for E7 to interact with nucleolar proteins involved in this process. GST-pull down and co-immunoprecipitation assays showed interactions between p14^ARF^, UBF1 and E7, although p14^ARF^ and E7 are not able to directly interact. Co-expression of a pRb-binding-deficient mutant (E7C24G) and p14^ARF^ resulted in EC24G nucleolar accumulation, but not in a significant higher activation of rDNA transcription, suggesting that the inactivation of pRb is involved in this phenomenon. Thus, p14^ARF^ fails to prevent E7-mediated UBF1 phosphorylation, but could facilitate nucleolar pRb inactivation by targeting E7 to the nucleolus. While others have reported that p19^ARF^, the mouse homologue of p14^ARF^, inhibits some functions of E7, we showed that E7 inhibits a p53-independent function of p14^ARF^. These results point to a mutually functional interaction between p14^ARF^ and E7 that might partly explain why the sustained p14^ARF^ expression observed in most cervical pre-malignant lesions and malignancies may be ineffective.

## Introduction

Human papillomaviruses (HPV) belonging to the high-risk (HR) or oncogenic group are major etiological agents for cervical cancer, other anogenital malignancies and, to a lesser extent, head and neck cancers [1]. Their transforming potential depends on deregulated expression of the viral oncoproteins E6 and E7. These proteins function through interactions with host regulatory proteins, most of which are involved in cell cycle progression, thus enabling the virus to overcome negative regulatory mechanisms [2]. One of the best-documented functions of HR-HPV E6 and E7 is the binding and degradation of the tumor suppressor proteins p53 and pRb, respectively. PRb proteolysis leads to the activation of E2F1-responsive genes, allowing cells to progress to S-phase. In normal proliferating cells this molecular switch is usually activated by the cyclin-dependent kinases CDK4/6, which induce pRb phosphorylation and inactivation. E7-positive cells strongly express the tumor suppressor p16^INK4a^ that inhibits cell cycle progression by inactivating CDK4/6. However, E2F1 is released in these cells by E7-mediated pRb degradation rather than CDK4/6 phosphorylation, thereby abrogating the growth-inhibitory functions of p16^INK4a^ [2]. P16^INK4a^ is encoded by the *INK4a/ARF* locus on human chromosome region 9p21, that also encodes p14^ARF^, another tumor suppressor protein [3]. Expression of p16^INK4a^ and inhibition of its functions have been widely explored in the context of high-risk HPV infection, and p16^INK4a^ is now considered as a useful indirect marker of high-grade cervical lesions [4]. In contrast, few studies have focused on p14^ARF^, even though p14^ARF^ expression is found in most cervical pre-malignant lesions and malignancies [5]–[8].

P14^ARF^, a predominantly nucleolar protein, is considered to be one of the most important oncogenic stress sensors [3], [9]. The main function of p14^ARF^ is to arrest the cell cycle in response to oncogenic stress, in a p53-dependent manner. P14^ARF^ inactivates the E3-ubiquitin ligase MDM2, a negative regulator of p53, leading to p53 stabilization and activation of p53-responsive genes [3]. In addition to MDM2 inactivation, p14^ARF^ stimulates p53 acetylation through hAda3, a component of histone acetyltransferase (HAT) complexes required for the full transcriptional activity of p53 [10]. The high-risk HPV oncoprotein E6 inactivates the p14^ARF^-p53 pathway by targeting p53 functions through several mechanisms. The most important of these mechanisms is probably E6-induced p53 degradation, through recruitment of the cellular E3-ubiquitin ligase E6AP [2]. P14^ARF^-dependent inhibition of MDM2 is inefficient in this case, as E6 acts independently of MDM2. E6 also uses indirect strategies, involving transcriptional co-activators (HAT or components of HAT complexes), to induce loss of p53 function. Thus, E6 inhibits p300-mediated p53 acetylation, leading to repression of p53-dependent gene activation [11]. More recently, it was shown that E6 induces hADA3 degradation and destabilization of HAT TIP60, a factor involved in p53-directed proapoptotic pathways, thereby also contributing to p14^ARF^-p53 pathway inactivation [12]–[13].

Besides these p53-dependent activities, there is growing evidence that p14^ARF^ also displays p53-independent biological activities that regulate not only cell growth but also apoptosis, angiogenesis, tumor cell migration and senescence [3], [9]. These functions are mainly achieved by inactivation of multiple cellular partners, through subcellular delocalization, stabilization or degradation. One p53-independent function of ARF is to inhibit the synthesis and processing of ribosomal RNA (rRNA) [14]–[19]. Ribosome biogenesis, which mainly takes place in the nucleolus, is a fundamental and complex process tightly coupled to cell growth and proliferation and usually upregulated in cancers and transformed cells [20]–[21]. Activation of rDNA transcription requires RNA polymerase I (Pol I) and several associated factors, including upstream binding factor 1 (UBF1), a key activator. UBF1, together with another Pol I-specific factor named SL1 that contains TATA-binding protein (TBP) and TBP-associated factors (TAF_I_ proteins), are involved in the assembly of the pre-initiation complex on the rDNA promoter [21]. The rate of ribosome biogenesis is regulated in part by mechanisms that control cell proliferation. Indeed, E2F1, c-Myc and epidermal growth factor enhance rDNA gene transcription, whereas cell-cycle inhibitors such as pRb and p53 repress it [22]–[25]. ARF (p14^ARF^ and p19^ARF^, the mouse homolog of p14^ARF^) inhibits ribosome biogenesis at several steps. P14^ARF^ can repress rDNA transcription by binding the rDNA promoter, interfering with UBF1 phosphorylation [14], [26], and inhibiting the nucleolar localization of the RNA polymerase I transcription termination factor TTF-1 [18]. ARF retards the processing of precursor rRNA, at least in part through its interaction with nucleophosmin (NPM), thereby reducing ribosome synthesis and subsequent cell proliferation [3], [9], [16]. ARF also inhibits polysome formation and protein translation [15]. Moreover, recent work has shown that ARF also inhibits the nucleolar localization of the RNA helicase DDX5, preventing its positive regulation of ribosome biogenesis [19].

Whereas the p53-dependent functions of p14^ARF^ are known to be inactivated by E6, the roles of the p53-independent activities of p14^ARF^ in HR E6/E7-expressing cells are unclear. P14^ARF^ is detectable in most cervical pre-malignant lesions and malignancies, in which ribosome biogenesis is thought to be upregulated, suggesting that the inhibitory function of p14^ARF^ is inefficient. Because E7 degrades pRb, a negative regulator of ribosome biogenesis, we suspected that E7 might engage in a functional relationship with p14^ARF^ during rDNA transcription.

Here, using luciferase assays, we show that E7 stimulates UBF1-driven transcription and inhibits p14^ARF^-mediated transcriptional repression of the *rRNA* gene promoter. E7 expression enhanced UBF1 phosphorylation, thus repressing the control exerted by p14^ARF^ on this fundamental RNA Pol I-transcription factor. Surprisingly, the transactivating effect of E7 was enhanced in p14^ARF^-expressing cells. One possible explanation is that the accumulation of E7 in the nucleolus upon p14^ARF^ expression may result in direct interactions between E7 and nucleolar factors involved in rRNA gene promoter regulation, such as UBF1 and nucleolar pRb. These findings show for the first time that E7 can inhibit a p53-independent function of p14^ARF^.

## Results

### E7 inhibits p14^ARF^-mediated repression of the rRNA gene promoter

**Figure 1:**
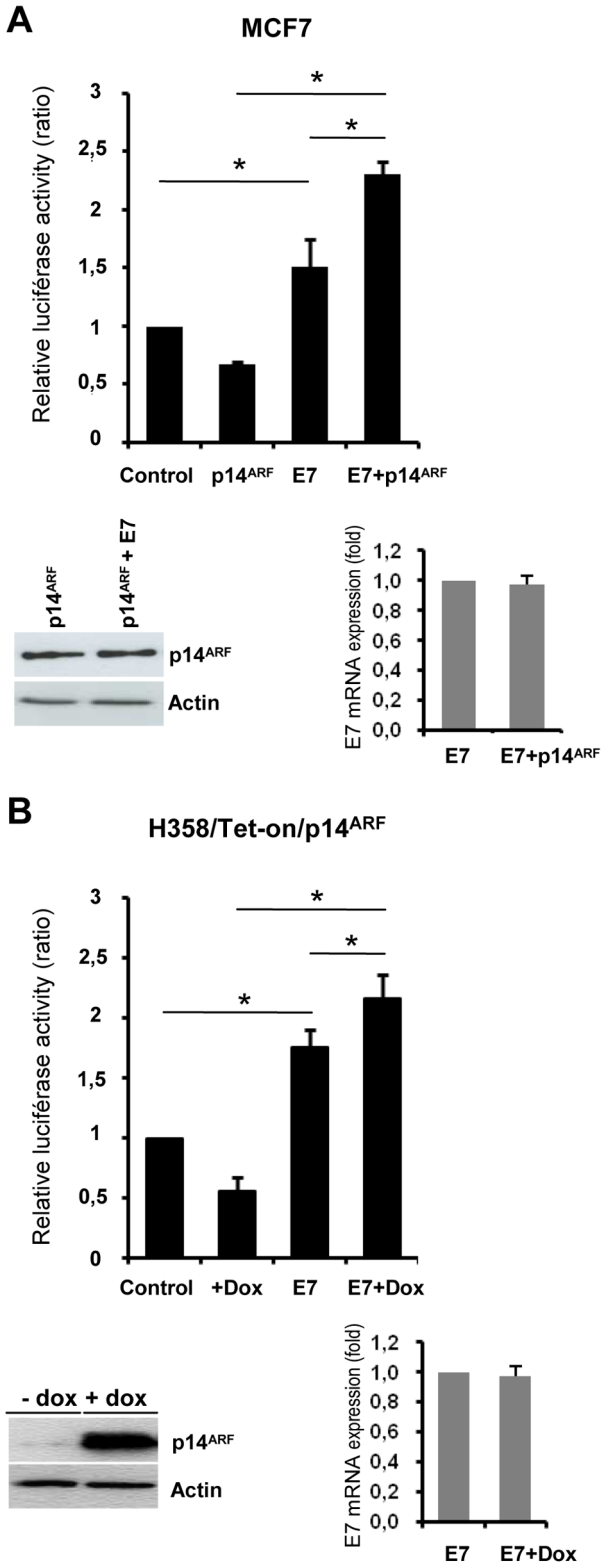
E7 inhibits p14^ARF^-mediated repression of the rRNA gene promoter. (A), rDNA promoter activity in MCF7 cells. The pHrDNA-IRES-Luc plasmid along with the *Renilla* internal control pRLTK, pcDNA3.1 (control), pcDNA3.1-p14ARF (p14^ARF^) and/or pJ4Ω16E7 (E7), were transiently cotransfected with Fugene 6 reagent. (B), rDNA promoter activity in H358/Tet-On/p14ARF cells. Cells were transduced with LXSN16E7 (E7) or with an empty retroviral vector (LXSN) as control. After selection with G418, cells were cultured with or without 1 mg/ml doxycyclin (Dox), then transiently transfected with pHrDNA-IRES-Luc together with the internal control pRLTK with TFX reagent. At 48 h, firefly and *Renilla* luciferase activity were measured. Reporter activity was calculated as the ratio of firefly luciferase activity to reference *Renilla* luciferase activity, and normalized so that luciferase activity in control vector-transfected cells equaled 1. Expression of p14^ARF^ and E7 was monitored by western blot and by quantitative real time RT-PCR, respectively. The data are representative of three independent experiments and are the means of triplicate samples (+/− SD, *p<0.05).

To determine whether E7 could interfere with rDNA transcription and p14^ARF^-transcriptional repression of the rDNA promoter, we performed luciferase reporter gene assays in multiple independent replicate experiments. We used an rDNA promoter construct (pHrDNA-IRES-Luc) spanning the core and upstream control element (UCE) regions that function synergistically to recruit the RNA Pol I transcription complex [27]. MCF7 cells (p14^ARF−/−^) were transiently transfected with pHrDNA-IRES-Luc, together with the *Renilla* control vector pRLTK, E7 and/or p14^ARF^ expression vectors, or with empty vectors (control). p14^ARF^ and E7 expression were controlled by western blot and qRT-PCR, respectively (Figure 1A). As previously shown [14], p14^ARF^ repressed rDNA promoter activation (Figure 1A). In contrast, RNA Pol I-dependent activity in MCF7 cells increased upon E7 expression even when p14^ARF^ was co-expressed. Thus, E7 prevents the ability of p14^ARF^ to repress the rDNA promoter. Similar results were obtained in a p53-deficient cell line (H358), indicating that the effects were p53-independent (data not shown).

Unexpectedly, promoter activity was significantly higher when E7 and p14^ARF^ were co-expressed in MCF7 (Figure 1A) and H358 cells (data not shown), by comparison with E7 expression alone (p<0,05), whereas no significant difference was observed in expression levels of E7 mRNA. To explore further, a H358/TetOn/p14^ARF^ inducible clone (clone 19) [28] was transduced with a retroviral vector expressing HPV16 E7 (LXSNE7) or with an empty vector (LXSN, control) and was selected with G418 for 10 days. Again, p14^ARF^ expression, induced by doxycycline treatment, was associated with an increase in the E7-enhanced activity of the rDNA promoter (Figure 1B). Taken together, these results suggested that p14^ARF^ is not dispensable to the action of HPV16 E7 on rDNA transcription but might facilitate it.

### pRb inactivation is involved in the increased rDNA promoter activity upon E7 and E7C24G co-expression

**Figure 2:**
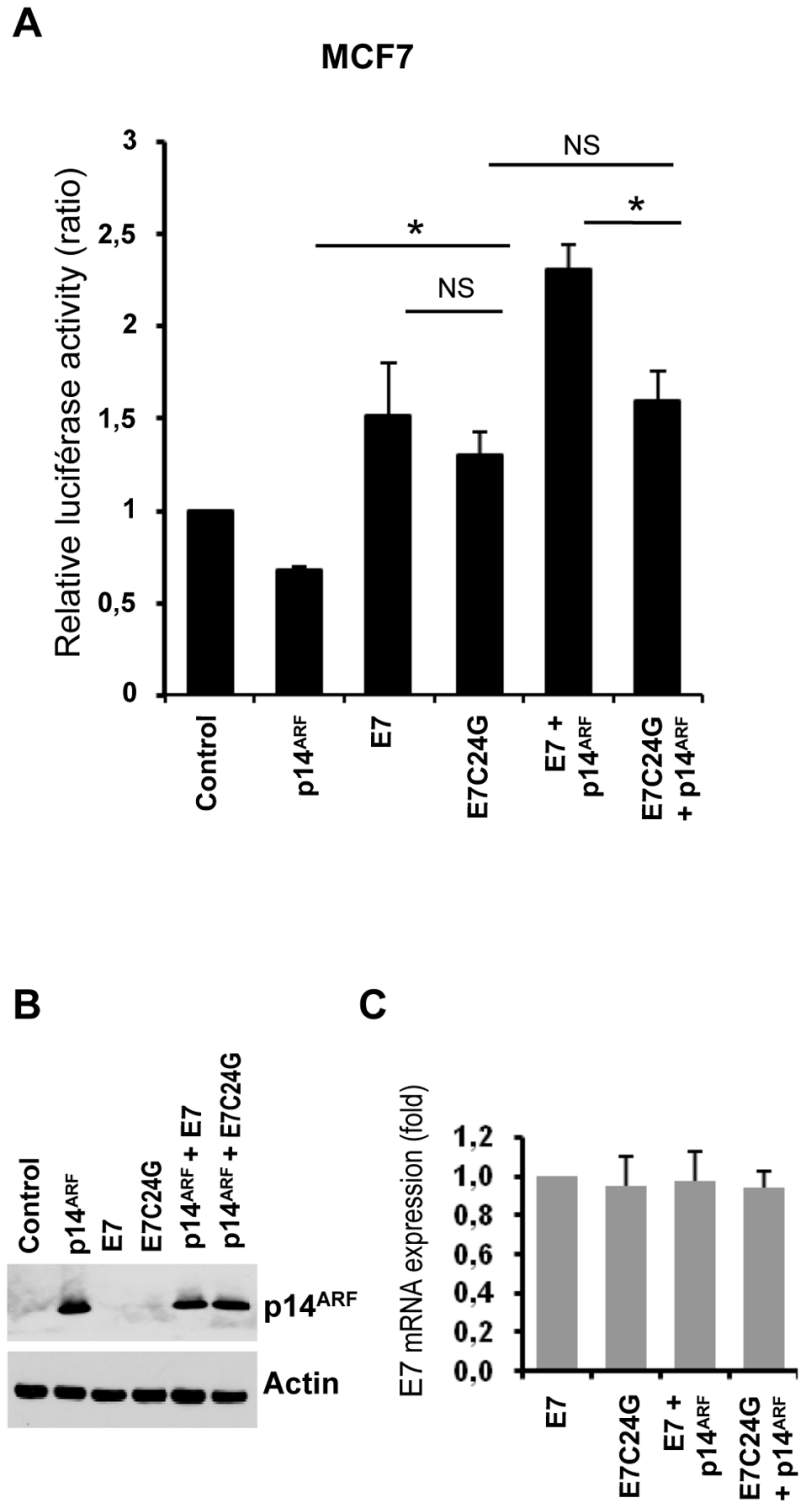
Role of pRb inactivation in the effect of E7 on the rDNA gene promoter. The pHrDNA-IRES-Luc plasmid and the internal control pRLTK were cotransfected into MCF7 cells along with pcDNA3.1(Control) and the indicated expression vectors. At 48 h, both firefly and *Renilla* luciferase activity were measured. Reporter activity was calculated as the ratio of firefly luciferase activity to reference *Renilla* luciferase activity, and normalized so that that luciferase activity in control vector-transfected cells equaled 1. The data are representative of three independent experiments and are the means of triplicate samples (+/− SD, *p<0,05). Expression of p14^ARF^ and E7 (E7 or E7C24G) was monitored by western blot (B), and quantitative real time RT-PCR (C), respectively.

As pRb modulates ribosome biogenesis by inhibiting rDNA transcription [24], we wondered whether the effect of E7 on rDNA transcription was due solely to E7-induced pRb inactivation. We thus compared the transcriptional activities of E7 and a pRb-binding-deficient mutant (E7C24G) in MCF7 cells, by using luciferase assays (Figure 2A). p14^ARF^ and E7 or E7C24G expression were controlled by western blot and qRT-PCR, respectively (Figure 2B and 2C). We found that E7C24G expression enhanced rDNA promoter activity, albeit less than E7 did, but this difference is not significant (p>0,99). E7C24G also inhibited p14^ARF^ repression. However, the rDNA promoter activity was not significantly enhanced in cells expressing E7C24G and p14^ARF^, compared to cells expressing E7C24G alone (p = 0,0053). It suggests that the inactivation of pRb is involved in the increase of rDNA promoter activity observed upon E7 and p14^ARF^ co-expression. These results indicated that pRb inactivation contributed to the E7-mediated stimulation of rDNA transcription but did not entirely account for the E7-dependent effects.

### E7 enhances UBF-driven transcription on the rDNA gene promoter

**Figure 3:**
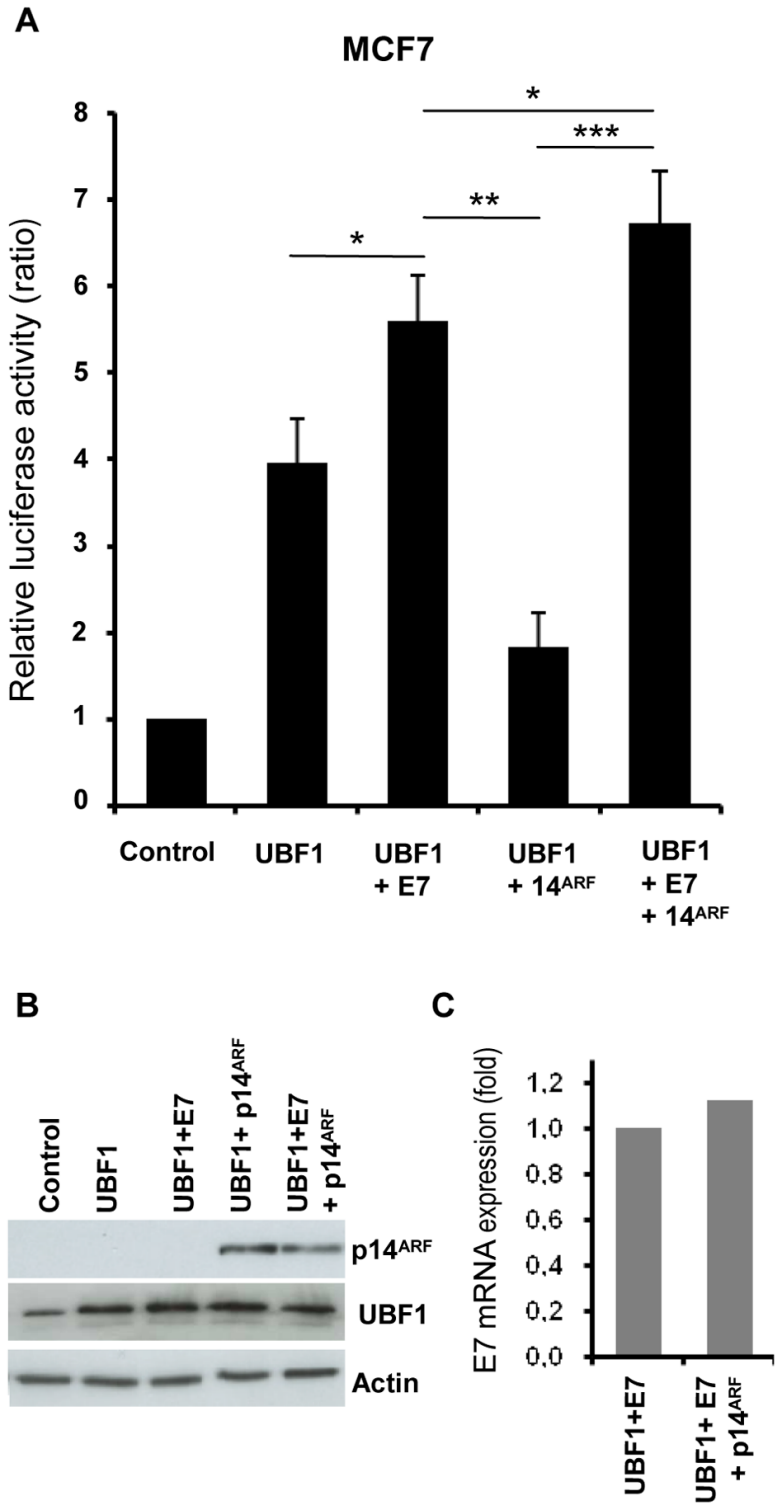
E7 enhances UBF1-driven transcription on the rDNA gene promoter. The pHrDNA-IRES-Luc plasmid and the internal control pRLTK were cotransfected into MCF7 cells along with pcDNA3.1(Control) and the indicated expression vectors. After 48 h, both firefly and *Renilla* luciferase activity were measured. Reporter activity was calculated as the ratio of firefly luciferase activity to reference *Renilla* luciferase activity, and normalized so that that luciferase activity in control vector transfected cells equaled 1. The data are representative of three independent experiments and are the means of triplicate samples +/− SD (*p<0.05, **p<0.01, ***p<0.001). Expression of p14^ARF^ and E7 was monitored by western blot (B), and quantitative real time RT-PCR (C), respectively.

pRb and p14^ARF^ inhibit rDNA transcription by interfering with the transactivator UBF1 [14], [29]. To further explore the impact of E7 on rDNA transcription, luciferase assays were performed with MCF7 cells transiently co-transfected with E7 and/or p14^ARF^ and UBF1 expression vectors. As shown in Figure 3, promoter activity was enhanced upon UBF1 overexpression (UBF1) and further enhanced in cells coexpressing UBF1 and E7 (UBF1+E7). These results indicated that E7 transactivated UBF1-driven transcription. Moreover, upon E7 expression, the repressive effect of p14^ARF^ was abrogated and promoter activity was significantly enhanced. Together, these data suggested that E7 stimulated rDNA transcription and inhibits p14^ARF^ by targeting UBF1.

### HPV16 E7 expression increases UBF1 phosphorylation and inhibits the effects of p14^ARF^

**Figure 4:**
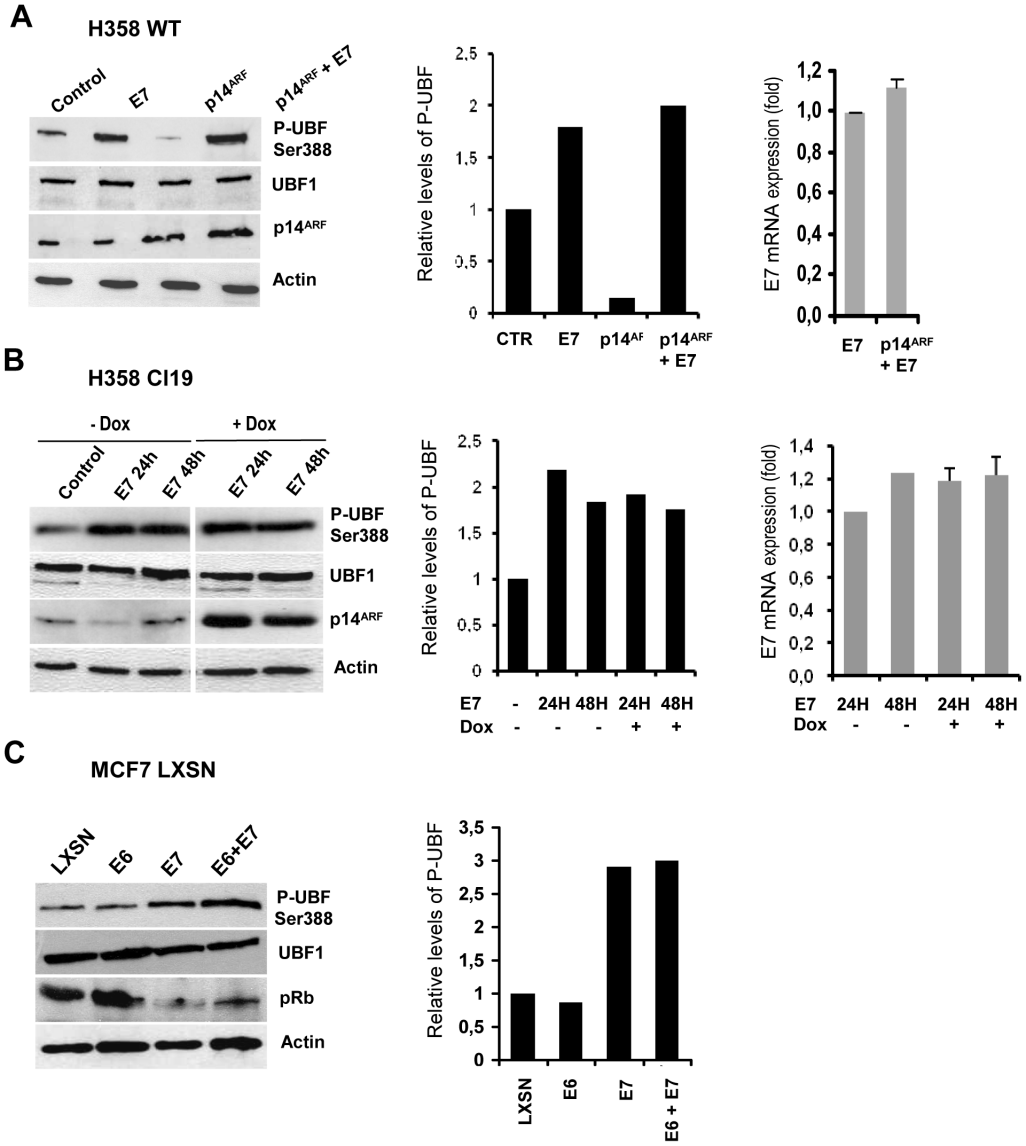
HPV16 E7 expression stimulates UBF1 phosphorylation. (A), Western blot analysis of H358 WT cells transiently transfected with the pcDNA3.1 (Control), pJ4Ω16E7 (E7) or pcDNA3.1-p14ARF (p14^ARF^) expression vector, or both and probed with antibodies to UBF, phosphorylated UBF (P-UBF Ser 388), p14^ARF^ and actin (as a loading control). (B), Western blot analysis of H358 Cl19 cells cultured with (+Dox) or without (-Dox) 1 mg/ml doxycyclin, transfected with pJ4Ω16E7 (E7), and probed with the indicated antibodies. (C), Western blot analysis of MCF7 cells transduced with LXSN, LXSNE7 (E7), LXSNE6 (E6), and LXSNE6E7 (E6+E7), selected with G418, and probed with the indicated antibodies. Quantification of western blots was done by measuring the relative intensity of the bands compared to internal controls (Actin), the values given are in arbitrary units. Expression of E7 was monitored by quantitative real time RT-PCR. Western blot and qRT-PCR are representative of three experiments at least.

Modulation of UBF1 activity by phosphorylation plays a pivotal role in cell-cycle-dependent regulation of rRNA synthesis [20]. Phosphorylation of UBF1 at serine 388, mediated by G1/S CDK2/cyclin, increases the interaction between RNA Pol I and activation of rDNA transcription [30]–[31]. The inhibition of rDNA transcription mediated by p14^ARF^ is associated with the interaction of p14^ARF^ and UBF1 and with subsequent UBF1 hypophosphorylation at serine 388 [14]. Since E7 stimulates CDK2 activity [32], we speculated that E7 could induce UBF1 phosphorylation at serine 388. Thus, we examined the phosphorylation status of UBF1 Ser 388 upon exogenous expression of E7 and/or p14^ARF^ (Figure 4). Using transient transfection assays in H358 cells (Figure 4A) and MCF7 cells (data not shown), we found that E7 enhanced UBF1 Ser 388 phosphorylation and circumvented the inhibiting effects of p14^ARF^, without affecting total UBF levels (Figure 4A). Similar results were obtained with H358/TetOn/p14^ARF^ cells induced by doxycyclin 24 h and 48 h after transfection with an E7 expression vector (Figure 4B). However, there was no significant difference in levels of phosphorylated UBF1 in cells expressing E7, compared to cells expressing E7 and p14^ARF^. Similar results were obtained with the E7C24G mutant (Figure S1A and S1B), suggesting that pRb inactivation is not involved in the UBF1 phosphorylation induced by E7. Thus, the level of phosphorylated UBF1 induced by E7 is not responsible for the increase in the E7-enhanced activity of the rDNA promoter observed upon p14^ARF^ co-expression, compared to E7 expression alone.

Next, we used cells stably expressing E7 and/or E6. MCF7 cells were transduced with LXSN-based retroviruses containing HPV16 *E7* alone (LXSNE7), HPV16 *E6* alone (LXSNE6), both HPV16 *E6* and *E7* (LXSNE6E7), or no insert (LXSN), and were selected for 10 days. Expression of E7 was confirmed by real time RT-PCR (data not shown) and pRb detection using western blot. Levels of Ser388-P-UBF1 were analysed by Western blot, (Figure 4C). E6 expression did not modify UBF1 phosphorylation on Ser388, whereas expression of E7 and of E6+E7 increased Ser388-P-UBF1 levels.

Together, these results suggested that the increased levels of Ser388-P-UBF1 induced by E7 contributed to the mechanism by which E7 stimulates rDNA transcription and suppresses p14^ARF^-dependent inhibition. However it does not account for the increased activity of the rDNA promoter observed upon E7 and p14^ARF^ co-expression.

### rDNA promoter activity and level of UBF phosphorylation upon E7 and/or p14^ARF^ depletion in the HPV16 positive CaSki cervical carcinoma line

**Figure 5:**
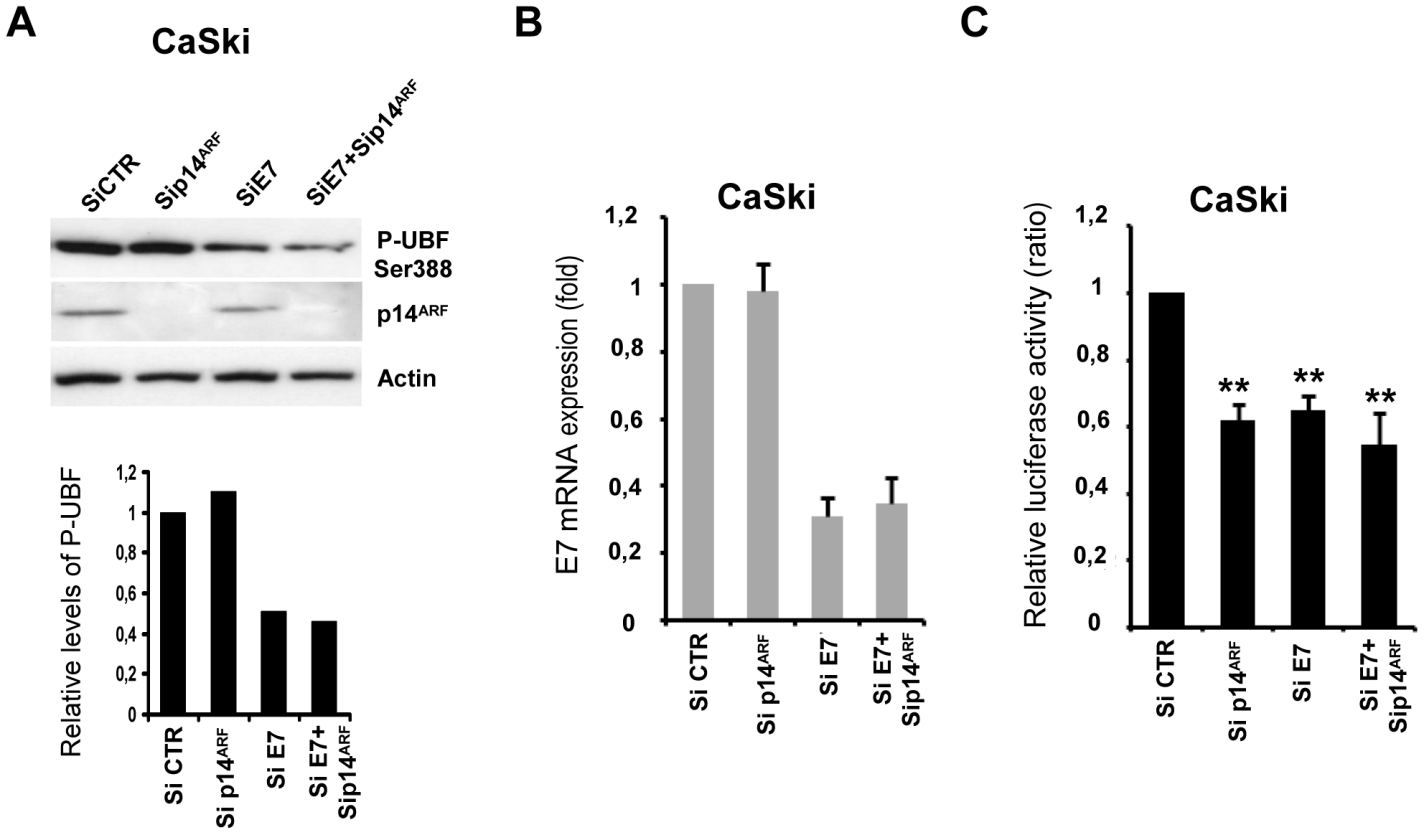
rDNA promoter activity and level of UBF phosphorylation upon E7 and/or p14^ARF^ depletion in the HPV16 positive CaSki cervical carcinoma line. E7 and/or p14^ARF^ were depleted in the HPV16 CaSki cervical carcinoma cell line using siRNA and Lipofectamine 2000 reagent. CaSki cells were transiently transfected with the pHrDNA-IRES-Luc plasmid along with the *Renilla* internal control pRLTK, siRNA control (Si CTR), E7 siRNA and/or p14^ARF^ siRNA and lysed at 48 h. (A) Western blot analysis of CaSki cells extracts probed with antibodies to phosphorylated UBF (P-UBF Ser 388), p14^ARF^ and actin (as a loading control). Quantification of western blots was done by measuring the relative intensity of the bands compared to internal controls (Actin), the values given are in arbitrary units. (B) Expression of E7 mRNA assessed by quantitative real time RT-PCR. (C) rDNA promoter activity. Reporter activity was calculated as the ratio of firefly luciferase activity to reference *Renilla* luciferase activity, and normalized so that that luciferase activity in control vector transfected cells equaled 1. The data are representative of three independent experiments and are the means of triplicate samples +/− SD (**p<0.01).

To further investigate the role of E7 and p14^ARF^ in rDNA promoter activity and UBF1 phosphorylation, we used p14^ARF^ siRNA and/or E6/E7 siRNA to deplete the CaSki cervical carcinoma cell line, and performed luciferase reporter gene assays and western blot. The efficiency of the siRNAs was determined by western blot (p14^ARF^) or RT-PCR (E7) (Figure 5A, 5B). Depletion of E7 mRNA (65 to 70%) induced a significant decrease in rDNA promoter activity associated with a decrease in Ser388-P-UBF1 levels (Figures 5A and 5B). This could be caused by the loss of the E7 enhancing effect, but could also result from the repressive effect of endogenous p14^ARF^ in the absence of E7 expression in CaSki cells. If the latter hypothesis is correct, increased rDNA promoter activity and Ser388-P-UBF1 levels would be expected upon siRNA silencing of p14^ARF^. Inversely, p14^ARF^ depletion induced a significant decrease in rDNA promoter activity without affecting the levels of phosphorylated UBF1, arguing against this hypothesis (Figure 5A). P14ARF and E6/E7 depletion also resulted in a decrease in rDNA promoter activity, slightly - but not significantly - greater than depletion of p14^ARF^ or E7 alone did (Figure 5C). Moreover, it induced a decrease in Ser388-P-UBF1 levels comparable to that observed upon silencing of E7 alone (Figure 5B).

These findings confirm the involvement of E7 in rDNA promoter activity and in Ser388-UBF1 phosphorylation. They also indicate that the activity of the rDNA promoter is higher when p14^ARF^ and E7 are co-expressed endogenously, by comparison with E7 expression alone (siRNA p14^ARF^), a result apparently not explained by Ser388-P-UBF1 levels. Thus, it confirms the data previously observed in MCF7 and H358 cells upon exogenous co-expression of E7 and p14^ARF^ (Figure 1 and Figure 4).

### p14^ARF^ induces E7 to accumulate in the nucleolar compartment

**Figure 6:**
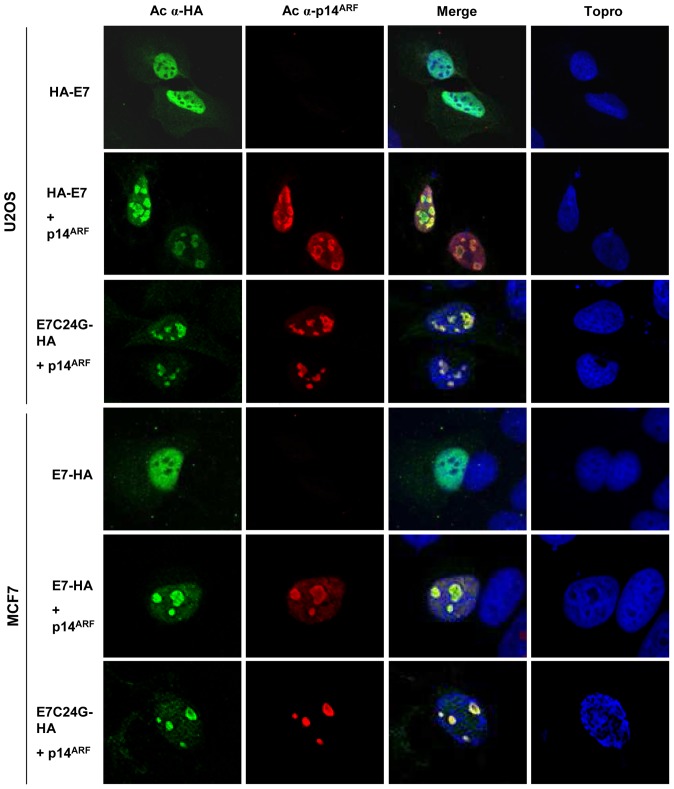
p14^ARF^ induces E7 and E7C24G to accumulate in the nucleolar compartment. MCF7 and U2OS cells were transfected with the pCMV16 E7- Flag/HA or pCMV16 E7C24G-Flag/HA vector and pcDNA3.1-p14ARF (p14^ARF^) by using the FuGENE 6 transfection reagent. At 48 h, samples were stained with anti-HA (green) and anti-p14^ARF^ (red) antibodies and analyzed by confocal laser scanning microscopy using a 60× objective. Cellular DNA was counterstained with Topro III. Superimposition of the figures confirms that nucleolar colocalization occurs only when p14^ARF^ is expressed.

rDNA transcription occurs in the nucleolus, where ARF is mainly located [3], [9]. A previous study has shown that p19^ARF^, the mouse homologue of p14^ARF^, can cause E7 to relocate from the nucleoplasm to the nucleolus in U2OS cells (p53^+/+^) [33]. As human ARF and mouse ARF exhibit some functional differences, we examined the subcellular localization of p14^ARF^ and E7 by indirect immunofluorescence in two different cell lines. As E7 was technically difficult to detect, we used a C-terminal HA-tagged E7 expression vector (pCMV16 E7-Flag/HA). This vector was transfected into U2OS and MCF7 cells, with or without a p14^ARF^ expression vector (pcDNA3-p14^ARF^). Cells expressing E7-HA alone displayed a predominantly nuclear distribution, excluding the nucleolus (Figure 6). However, in response to p14^ARF^ expression, E7 accumulated in the nucleolus and colocalized with p14^ARF^ in the granular region at the periphery of the nucleolar compartment. This nucleolar location could provide an explanation of the higher rDNA promoter activity upon E7 and p14^ARF^ coexpression. Since E7C24G did not exhibit the same effect on rDNA transcription when p14^ARF^ is coexpressed, we determined whether p14^ARF^ is able to induce the localization of E7C24G in the nucleolus. Like E7, the mutant displayed nucleolar accumulation when p14^ARF^ is coexpressed, but in any case, it cannot interact with nucleolar pRb.

### E7 interacts with UBF1 and p14^ARF^

**Figure 7:**
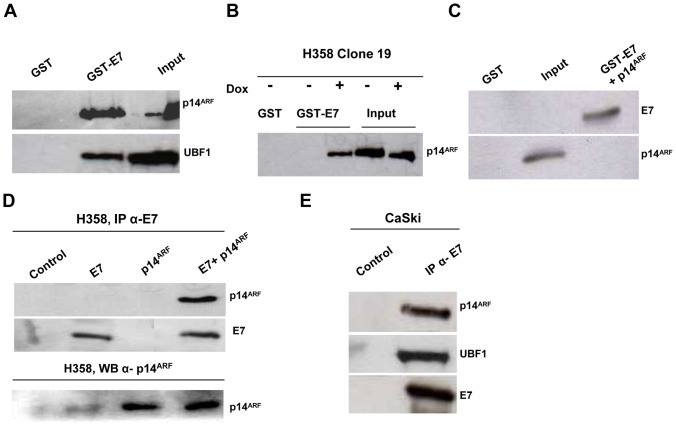
E7 interacts with p14^ARF^ and UBF1. (A), The GST-E7 fusion protein efficiently pulled down p14^ARF^ and UBF1. GST-E7 or GST alone was mixed with CaSki cell lysates. UBF1 and p14^ARF^ were detected by Western blot. (B), E7 interacts with UBF1 only when p14^ARF^ is expressed. GST-E7 or GST alone was added to H358 Cl19 cell lysates after culture with or without 1 mg/ml doxycyclin (Dox) for 24 hours to induce p14^ARF^ expression. UBF1 was detected by western blot. (C), Indirect interaction between E7 and p14^ARF^. Purified p14^ARF^ was incubated with GST-E7 or GST alone, immobilized on glutathione resin, and complexes were analyzed for the presence of p14^ARF^ by western blot. (D), E7 interacts with p14^ARF^ in H358 cells (p53−/−). Cells were transiently transfected with p14^ARF^ and E7 expression vectors. Lysates were immunoprecipitated with purified mouse IgG1 (control) or anti-HPV16-E7 antibodies, and immune complexes were detected with antibodies to p14^ARF^ and to HPV16-E7. (E), E7 interacts physiologically with p14^ARF^ and UBF1 in CaSki cells. Lysates were immunoprecipitated with purified mouse IgG1 (control) or anti-HPV16-E7 antibodies. Immune complexes were detected with antibodies against p14^ARF^, UBF1 and HPV16-E7.

E7 is known to modulate transcription by interacting with transcription factors or regulators [2]. The above findings strongly suggested that E7 can interact with UBF1 and/or p14^ARF^. To test this possibility, we performed GST pull-down and immunoprecipitation experiments. Purified recombinant GST-E7 and GST alone were mixed with CaSki cell lysates as a source of endogenous p14^ARF^ and UBF1. Precipitated complexes were examined by Western blot analysis, after extensive washes, for the presence of p14^ARF^ and UBF1. The GST-E7 fusion protein efficiently pulled down p14^ARF^ and UBF1, while the GST protein alone did not (Figure 7A). To determine the role of p14^ARF^, we performed GST pull-down assays with GST-E7, GST alone, and extracts from H358/TetOn/p14ARF cells induced with doxycycline. Interaction between UBF1 and E7 was only seen when p14^ARF^ was overexpressed (Figure 7B). To determine whether the interaction between p14^ARF^ and E7 was direct or mediated by other proteins present in the cell lysate, we performed a direct binding assay in which the two proteins were expressed and purified from bacteria. Soluble, purified p14^ARF^ was incubated with GST-E7 and GST alone, immobilized on glutathione resin, and complexes were examined for the presence of p14^ARF^. As shown in Figure 7C, no interaction was seen between p14^ARF^ and GST-E7, suggesting that p14^ARF^ and E7 do not interact directly.

We then examined interactions in H358 and CaSki cells in order to test the physiological relevance of the interactions between E7, p14^ARF^ and UBF1 observed in vitro. Co-immunoprecipitation experiments were carried out with extracts of H358 cells transiently transfected with a p14^ARF^ expression vector and/or an E7 expression vector, using anti-E7 antibodies (ED17 and 8C9) or mouse IgG1 as control. As shown in Figure 7D, p14^ARF^ was immunoprecipitated by anti-E7 antibodies but not by control IgG1 (Figure 7D). In CaSki cells, E7 co-precipitated with p14^ARF^ and UBF1, both of them endogenously expressed, demonstrating the relevance of these interactions in physiological conditions (Figure 7E). Moreover, endogenous E7 was also specifically immunoprecipitated by anti-p14^ARF^ antibodies (data not shown).

Together, these results suggest that p14ARF may form a complex with E7 and other proteins, thereby promoting nucleolar delocalization and UBF1-E7 interaction.

### E6/E7 expression in human cervical keratinocytes results in early p14^ARF^ expression and increased Ser388-P-UBF1 levels

**Figure 8:**
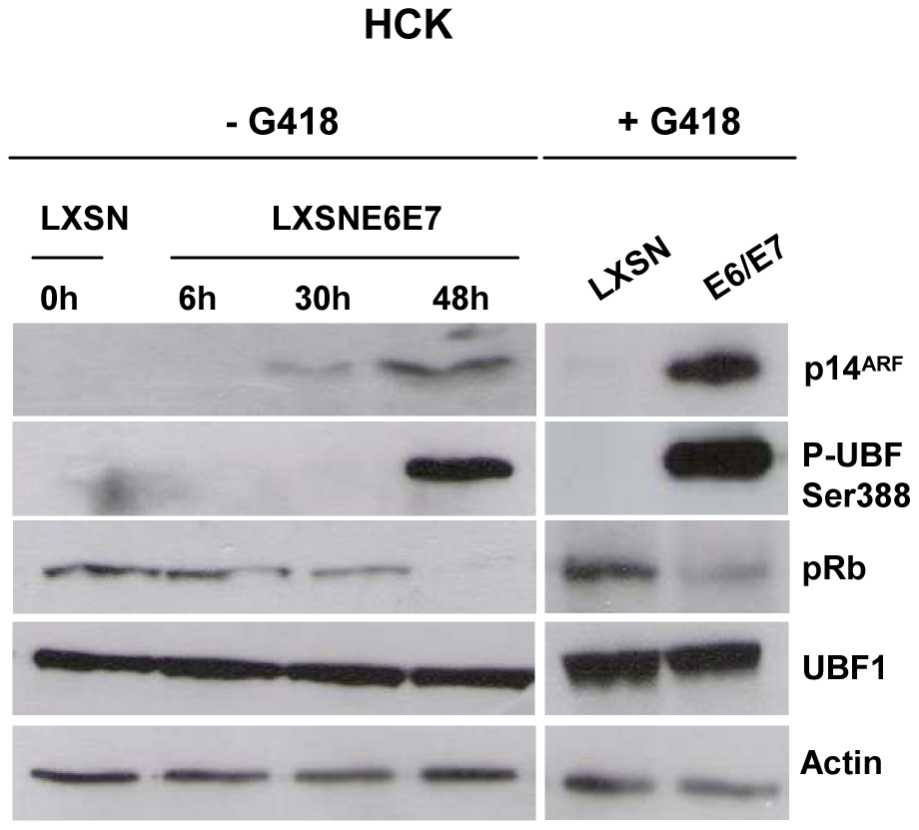
Expression of HPV16 E6/E7 in human cervical keratinocytes induces p14^ARF^ expression early after transduction and enhances UBF1 phosphorylation. Human keratinocytes were transduced with retroviral vectors encoding E6 and E7 (LXSNE6E7) or with an empty vector (LXSN), and were then cultured with or without G418. Protein extracts were analysed by Western blot at various times, using antibodies against p14^ARF^, UBF, p-UBF(Ser 388), actin and pRb.

Expression of p14^ARF^ has been detected in cervical cancers and in most neoplastic lesions induced by high-risk HPV [5]–[8]. To determine when p14^ARF^ might be induced after infection, we transduced pooled human primary cervical keratinocytes (HCK) with LXSN-based retroviruses containing no insert (LXSN) or HPV16 *E6+E7* (LXSNE6E7), and monitored p14^ARF^ expression. Expression of HPV16 E6/E7 was assessed by real-time RT-PCR (data not shown) and indirectly by Western blot (pRb). Levels of p14^ARF^ were measured by Western blot. P14^ARF^ induction was detectable 30 h post-transduction in HCK cells transduced with HPV16 E6+E7 and was associated with E7 expression confirmed by reduced levels of pRb (Figure 8). Increased levels of p14^ARF^ were observed after selection and 10 passages. These results confirmed that E6/E7 expression was sufficient to induce p14^ARF^ and that this induction occurred early after transduction, before HCK cell immortalization. Moreover, as observed with MCF7 and H358 cells, UBF1 Ser388 phosphorylation was enhanced in HCK cells upon E6/E7 expression, and in spite of p14^ARF^ induction.

## Discussion

Cells stimulated to proliferate during normal and pathological processes exhibit increased ribosome biogenesis. Production of rRNA is a major rate-limiting step in ribosome biogenesis, determining the cell's capacity to synthesize proteins that regulate its growth [20]–[21]. Whereas c-Myc and NPM exert positive control on rDNA synthesis, the tumor suppressors pRb, p53 and p14^ARF^ have the opposite effect [22]–[25], [34]. Thus, upregulation of rRNA synthesis can result from deregulation of either tumor suppressors or oncogenes, leading to uncontrolled cell growth and proliferation. In this study we showed that the viral oncoprotein HPV16 E7 stimulated rDNA promoter activity and prevented p14^ARF^ repression. This effect was p53-independent, as similar results were obtained in a p53-deficient cell line (H358). As E7 induces pRb degradation, E7 might be expected to deregulate pRb-dependent inhibition of rDNA transcription. To go further into the involvement of pRb degradation by E7, we used a pRb-binding-deficient E7 mutant (E7C24G). It appeared that the enhancing effect of E7 was not due solely to the loss of pRb, as E7C24G also enhanced rDNA promoter activity and inhibited p14^ARF^ repression.

Previous studies have shown that other oncogenic viruses, including SV40 and HCV, also activate rDNA transcription [35]–[37]. UBF1 is the main target for this viral-induced transcriptional activation. The SV40 large T antigen (LT) and the HCV non-structural protein NS5A induce an increase in UBF1 phosphorylation [38]–[39]. The underlying mechanisms involve kinase activity associated with SV40 LT and/or, in the case of HCV, upregulation of both cyclin D1 and CDK4 by NS5A. Furthermore, inhibition of rDNA transcription by p14^ARF^ is achieved both directly, through reduced UBF1 phosphorylation and recruitment on the transcription complex, and indirectly through p53 induction [14], [25]–[26]. Thus, these data prompted us to investigate UBF phosphorylation to further define the molecular mechanism involved in E7-mediated rDNA promoter activation. Our results showed that E7 and E7C24G expression both enhance Ser 388 phosphorylation of UBF1. UBF1 phosphorylation at Ser 388 that is required for setting up the initiation complex, RNA pol I recruitment, and activation of rDNA transcription, depends on CDK2/cyclin E and CDK2/cyclin A [31]. CDK2/cyclin E activity rises shortly before S-phase onset, and cell cycle progression into S phase involves CDK2/cyclin E and CDK2/cyclin A activation [40]. The increased UBF1 phosphorylation observed in cells expressing E7 could be explained in part by the increased levels of cyclin E and subsequent CDK2 activation, that reflect the effects of E7 on S phase entry [32], [41]–[42]. Interestingly, there was no significant difference in levels of phosphorylated UBF1 in cells expressing E7, compared to cells expressing E7 and p14^ARF^. Moreover, silencing of p14^ARF^ in CaSki cells did not affect levels of phosphorylated UBF1, but induced a decrease in rDNA promoter activity. These results demonstrate that the inhibitory action of p14^ARF^ on UBF phosphorylation and rDNA promoter activity are inefficient in E7-expressing cells in the context of HPV-induced malignancy.

Intriguingly, we also found that the E7-induced transcriptional activation of the rDNA promoter in MCF7 and H358 cells was moderately but significantly increased upon p14^ARF^ co-expression. We cannot exclude a slight difference in E7 expression at the protein level. However, the decrease in rDNA promoter activity upon silencing of p14^ARF^ in CaSki cells provides further evidence of this finding. This effect was not clearly observed with the E7C24G mutant, suggesting an involvement of pRb binding and degradation. We found no significant difference in levels of phosphorylated UBF1 in cells expressing E7 or E7C24G, compared to cells expressing E7 and p14^ARF^ or cells expressing E7C24G and p14^ARF^. Thus, a difference of phosphorylated UBF1 levels does not account for the increased activity of the rDNA promoter observed upon E7 and p14^ARF^ co-expression. Pan *et al* [33] showed that the introduction of the murine protein p19^ARF^ in ARF-deficient U2OS cells induced nucleolar localization of E7. Moreover, immunofluorescence and electron microscopy with immunogold staining revealed the presence of E7 within the nucleolus of the HPV16-positive cervical carcinoma cell line CaSki, that expresses endogenous p14^ARF^ [43]. Accordingly, we show that E7 accumulates in the nucleolar compartment of U2OS and MCF7 cells upon p14^ARF^ overexpression. The E7C24G mutant exhibited a similar nucleolar accumulation upon p14^ARF^ expression. Together, these results suggest that the nucleolar localization of E7 facilitates its enhancement of rDNA transcription by a mechanism involving pRb degradation. In the nucleolus, Rb directly represses transcription of the rRNA genes by binding to UBF1 and inhibiting its DNA binding activity [21], [24]. We provided evidence that E7 interacts with UBF1 and with p14^ARF^. These interactions are physiologically relevant, as they were also observed in immunoprecipitation experiments in CaSki cells. However, we detected no direct interaction between E7 and p14^ARF^, suggesting that the interaction occurs through a protein complex, probably involving UBF1. Thus, a possible explanation of the increased E7-activation of rDNA transcription is that the nucleolar accumulation of E7 could allow E7 to inhibit the binding of pRb to UBF1 and facilitate the degradation of nucleolar pRb. SV40 LT, that shares both structural and functional features with E7, is recruited to the nucleolar rDNA promoter through direct protein-protein interactions with the SL1 initiation factor and the formation of a stable UBF1-SL1 complex [44]. This interaction with TBP-TAF complexes is part of the mechanism by which SV40 LT activates rDNA transcription [35]. E7 has also been shown to interact with TBP and TAF110, a TBP-associated factor [45]-[46], raising the possibility that its nucleolar localization could also allow E7 to interact with SL1 on the rDNA promoter. The idea that p14^ARF^-induced nucleolar localization of E7 can facilitate its enhancement of rDNA transcription could be somewhat paradoxical, since p14^ARF^ alone inhibits rDNA transcription and displays tumor suppressor activities. Pan *et al* [33] have provided evidence that p19^ARF^ can inhibit two functional properties of E7, namely pRb proteolysis and stimulation of DNA replication. They suggested that this effect could rely on p19^ARF^-dependent nucleolar sequestration of E7. Recent data indicate that several functions of adenovirus oncoprotein E1A, which shares certain features with E7 and SV40 LT, are inhibited by p14^ARF^ [47]. The authors linked the nucleolar localization of E1A and E7 to the loss of their function. As we show here, E7 may in turn inhibit a nucleolar function of p14^ARF^ and nucleolar localization of E7 may facilitate its enhancement of rDNA transcription. Although puzzling, these results are not be mutually exclusive. Indeed, such relationships have already been described between ARF and another protein, the nucleophosmine NPM. NPM is a multifunctional shuttling protein involved in ribosome biogenesis, regulation of centrosome duplication, DNA repair, RNA transcription and apoptosis [48]. NPM targets ARF to the nucleolus and this localization has been associated with inhibition of several ARF functions [48]–[49]. However, nucleolar localization of ARF induces its stabilization and is linked to specific functions, including negative regulation of ribosome biogenesis [14], [16]–[17]. Conversely, ARF has been reported to affect the function of NPM by inducing NPM SUMOylation, degradation, impeding its shuttling, and inhibiting rRNA processing [3], [17], [48]. E7 and ARF, like NPM and ARF, display antagonistic properties, but the nucleolar localization of E7 induced by ARF seems to potentiate the enhancing effect of E7 on rDNA transcription. The puzzling idea that a tumor suppressor could exert some oncogenic activity under certain circumstances has always been explored. Humbey *et al* [50] have previously shown that p14^ARF^ may have a tumor –promoting activity that could limit the progression of some tumors, such as lymphoma. Moreover, McLaughlin-Drubin *et al* [51] have recently evidenced that p16^INK4A^, a tumor suppressor highly expressed in response to E7, displays an oncogenic activity in HPV16+ cervical cancer cells depending on inhibition of CDK4/CDK6 and cellular context. Our results do not prove that p14^ARF^ could exert an oncogenic function by itself. However, it is conceivable that the abundance of each protein may modulate the nature of their cellular partners, as well as their localization and functions.

In addition to its negative control of rRNA synthesis, ARF can also inhibit rRNA processing and rRNA nuclear export. These functions can be achieved through ARF binding to NPM [17], [19], [48] and through ARF-induced nucleolar localization of the RNA Pol I transcription termination factor TTF-1 [18]. Recently, E7 was shown to upregulate NPM levels in E7-expressing differentiating cells and proliferating cells [52]. It is tempting to speculate that this upregulation could contribute to abrogating p14^ARF^ control of ribosome biogenesis.

The HPV replicative cycle is unusual. Whereas HPV infects cells of the basal layer of stratified squamous epithelia, new virions can only be produced by differentiated cells of the suprabasal layers which, in physiological conditions, have exited the cell cycle [2]. HPV replication being tightly dependent on cellular DNA replication, the proliferative capacity of infected cells must be uncoupled from their differentiation. Maintenance of S-phase competence in differentiated cells requires the abrogation of cell-cycle checkpoints, which is achieved by proteins E6 and E7. Interestingly, however, E6 and E7 expression is only detected in suprabasal layers during the productive viral cycle. ARF is considered to be a potential nucleolar integrator of growth signals, and Apicelli *et al* [17] recently proposed that basal ARF acts as a monitor of steady-state ribosome biogenesis and growth. ARF can be induced by protein E7 from the cottontail rabbit in E6/E7- or E7-immortalized rabbit keratinocytes [53] and by HPV16 E7 in E6/E7-expressing keratinocytes [52]. Our data show that E7 expression can induce p14^ARF^ early after transduction of primary cervical keratinocytes, before their immortalization. Thus, immortalization is not essential for E7-dependent p14^ARF^ induction. E7 could induce p14^ARF^ during productive viral cycling in suprabasal cell layers. Our results are in agreement with those of Garcia *et al* [54], who showed that ARF could be induced by viral infection and proposed that ARF might act as a viral stress sensor, restricting virus infection in a p53-independent manner. Here we observed increased levels of Ser388-phosphorylated UBF1 upon E7 expression and p14^ARF^ induction in cervical keratinocytes. It suggests that E7 could stimulate rDNA transcription during the HPV replicative cycle. Nucleolar E7 accumulation can limit nuclear pRb degradation [33] but could also lead to degradation of nucleolar pRb. Moreover, we cannot exclude the possibility that E7 has other nucleolar functions, in addition to facilitating rDNA transcription. Many viruses are known to exploit the nucleolar compartment in order to facilitate their replication [55]. Thus, targeting of viral proteins to the nucleolus may also play a role in viral replication processes.

In conclusion, we show that HPV16 protein E7 stimulates rDNA transcription and inhibits a p53-independent function of p14^ARF^. Moreover, the nucleolar localization of E7 facilitates its enhancement on rDNA transcription by a mechanism independent of UBF1 phosphorylation but dependent of pRb-E7 interaction. Although stimulation of rDNA transcription is probably involved in the HPV replicative cycle, it could also contribute to the oncogenicity of high-risk HPV. Growth-stimulating signals triggered by the E7 oncoprotein and growth-suppressive signals derived from the p14^ARF^ tumor suppressor pathway oppose one another and could influence the outcome of infection. However, the sustained p14^ARF^ expression observed in most cervical pre-malignant lesions and malignancies suggests that p14^ARF^ is ineffective in these cells. Additional experiments will be needed to determine whether the other p53-independent functions of p14^ARF^ are similarly inhibited by E7.

## Materials and Methods

### Ethics statement

Oral informed consent was obtained from the patients providing fresh cervical tissue by the Gynecology Unit of Poitiers University Hospital (Pr G Magnin). This study was approved by the institutional ethic committee (Centre Hospitalier Universitaire, Poitiers).

### Cell culture and establishment of cell lines

MCF7 human breast adenocarcinoma cells (ARF-null) and U2OS human osteosarcoma cells (ARF-null) were grown in Dulbecco's modified Eagle's medium (DMEM). H358 human bronchoalveolar carcinoma cells (ARF-deficient, p53-null) and CaSki, a HPV16-positive human cervical carcinoma cell line, were grown in RPMI 1640 medium (Invitrogen). The H358/Tet-On/p14^ARF^ inducible clone (H358 clone 19), kindly provided by B. Eymin, was obtained by using a modified doxycyclin-regulated inducible expression system (Tet-On system, Clontech), as previously described [28]. P14^ARF^ expression was induced in the presence of 1 µg/ml doxycyclin (Sigma-Aldrich). The media were supplemented with 10% FBS and antibiotics (100 U penicillin/ml, 100 U/ml streptomycin) (Invitrogen) and the cells were cultured at 37°C with 5% CO2.

Primary human ectocervical keratinocytes (HEK) were isolated from fresh cervical tissue obtained during hysterectomy for benign uterine diseases after informed consent. The epidermal layer was separated from the dermis with 25 U/ml dispase supplemented with 5 µl/ml gentamicin overnight at 4°C. Keratinocytes were then isolated from the epidermal layer with standard trypsination procedures and cultured in serum-free keratinocyte medium supplemented with insulin, epidermal growth factor and fibroblast growth factor (Defined Keratinocyte-SFM, Invitrogen). After reaching 85% confluence, the cells were trypsinized, expanded once, and frozen. Pooled keratinocytes were obtained from vials of keratinocytes derived from 5 individuals.

Cell lines stably expressing E7-HPV16 and/or E6-HPV16 were established by using Phoenix Ampho packaging cells (Orbigen) and LXSN amphotrophic retroviral constructs (LXSN, LXSN16E6, LXSN16E7, LXSN16E6E7) kindly provided by Dr D. Galloway. Phoenix cells were cultured in DMEM supplemented with 10% FBS and 2 mM L-glutamine. Twenty-four hours before transfection, 1.5 to 2×10^6^ cells were cultured in T25 flasks. Transfection with LXSN constructs was performed with Fugene 6 (Roche), and recombinant retroviruses were prepared following the manufacturer's instructions (Orbigen). Second-passage HFK were pooled and infected after thawing with recombinant retroviruses mixed with Polybrene (4 ug/ml). Three days after infection, cells were selected with G418 (400 µg/ml). HPV16 E6 and E7 were detected by RT-PCR or immunoprecipitation, and the concentration of G418 was then reduced by half. MCF7 and H358 clone 19 were similarly transduced.

### Plasmids

The pcDNA3.1-p14ARF, pcDNA-UBF and pGST-p14ARF vectors are described in detail elsewhere [14], [26]. The luciferase construct (pHrDNA-IRES-Luc) was kindly provided by Dr S. Jacob [27]. pIRES-Luc was constructed by replacing the Kozac sequence of a pGL3-basic vector (Promega) with the IRES. pHrDNA-IRES-Luc was then obtained by cloning the human rRNA promoter sequence (−410/+314) comprising the UCE, the core promoter and part of the ETS1, upstream of IRES-Luc. pRLTK corresponds to the *Renilla* luciferase reporter driven by the HSV-TK promoter (Promega). pJ4Ω16E7 (E7) was kindly provided by Dr L Banks [56]. The pCMV16 E7-Flag/HA vector was obtained from Addgene (Addgene plasmid 13733). We also thank Dr A Baldwin for this construct [57]. pCMV16 E7C24G-Flag/HA was obtained by site-directed mutagenesis with the QuickChange kit (Stratagene). PGEX-E7WT (GST-E7) was kindly provided by Dr T Kouzarides. pcDNA3.1-16 E7C24G was obtained by cloning E7C24G from plasmid pGEX2T-HPV16 E7C24G into pcDNA3.1 at the EcoRI and BamHI restriction sites. All cloning products were verified by DNA sequencing.

### Transfections and luciferase assays

MCF7 and H358 cells were subcultured and seeded in six-well plates. After 24 h, cells were transfected with empty pcDNA3.1 (control), pcDNA3.1 expressing ARF (p14^ARF^) and/or pJ4Ω16E7 (E7), and/or pcDNA3.1-16 E7C24G, or pcDNA-UBF (0.5 or 1 µg) using the Fugene (FuGENE 6 Transfection Reagent, Roche) or TFX 50 (Promega) transfection reagents, according to the manufacturers' instructions. H358 cl19 cells transduced with LXSN or LXSNE7 and selected with G418 were treated with doxycyclin (1 mg/ml) for 48 h before transfection. All transfections were adjusted with empty vector DNA to contain the same total amount of DNA per well. Cells were harvested for dual luciferase assays, western blotting, immunoprecipitation or qPCR at 24 and/or 48 h after transfection.

CaSki cells were transfected using Lipofectamine 2000 (Invitrogen). Si RNA for p14^ARF^ (5′-GAACAUGGUGCGCAGGUU-C**TT**-3′) and for E7 (Si16E6/E7, 5′-UUAAAUGACAGCUCAGAGG-3′) [58] were custom made by Invitrogen. A scrambled SiRNA was used as control (Invitrogen). Expression of p14^ARF^ or inhibition was checked by immunoblot analysis. Expression of E7 or inhibition was checked by detection of pRb by western blot or qRT-PCR.

For luciferase assays, cells were transfected together with pHrDNA-IRES-Luc (200 ng), the pRLTK reference plasmid (10 ng) and the plasmids described above using the Fugene (FuGENE 6 Transfection Reagent, Roche) or TFX 50 (Promega) transfection reagents, according to the manufacturers' instructions. Forty-eight hours after transfection, cells were lysed with Passive Lysis Buffer and firefly and *Renilla* luciferase activities were determined using the Dual Luciferase Assay System (Promega) in a TD20/20 luminometer (Promega). Reporter activity was calculated as the ratio of firefly luciferase activity to reference *Renilla* luciferase activity, and normalized so that luciferase activity in control vector-transfected cells equaled 1. Within each experiment, transfections were done in triplicate, and the results reported are from three independent experiments.

### Western blot analysis

Cells were washed in phosphate-buffered saline (PBS), resuspended in lysis buffer (Tris-HCl 10 mM pH 7.5, NaCl 120 mM, EDTA 1 mM, dithiothreitol 1 mM, NP40 0.5%, sodium dodecyl sulfate (SDS) 0.1%, phenylmethylsulphonyl fluoride (PMSF) 1 mM, supplemented with a protease and phosphatase inhibitor cocktail (Sigma). Protein extracts were normalized for total protein concentration by BCA kit (Bicinchoninic Acid, SIGMA), resolved by SDS-PAGE (12 or 15%) and transferred to nitrocellulose. The membrane was blocked with 5% non-fat milk in Tris-buffered saline containing 0.1% Tween 20 (TBS-T) for 1 h and then incubated overnight at 4°C with primary antibodies diluted in blocking solution. The primary antibodies included antibodies against UBF (H300, Santa Cruz Biotechnology), phosphorylated UBF (pUBF-Ser388-R, Santa Cruz Biotechnology), p14ARF (C18, Santa Cruz Biotechnology), pRb (C15, Santa Cruz Biotechnology) and actin (AC-74, Sigma) or GAPDH (Sigma) for loading controls for cell lysates. The membrane was then washed three times with TBS-Tween and incubated with appropriate HRP-conjugated secondary antibodies for 1 h at room temperature. After washing five times with TBS-T, the membrane was developed with an enhanced chemiluminescence kit (ECL+ detection kit, Amersham, France) according to the manufacturer's instructions. Quantification of western blots was done by measuring the relative intensity of the bands compared to internal controls (Actin or GAPDH), the values given are in arbitrary units.

### Immunoprecipitation

Protein extracts were cleared by centrifugation (16,000 x g, 30 min, 4°C]. Supernatants were precleared with Protein-A/G-agarose by gently rotating at + 4°C for 30 min and immunoprecipitated overnight at + 4°C with the following primary antibodies: C18 anti-p14^ARF^ (Santa Cruz Biotechnology), anti-HPV16-E7 (8C9, Zymed, Invitrogen Ltd, and ED17, Santa Cruz Biotechnology) or mouse IgG1 as control. Immune complexes were purified by using Protein-A/G-agarose beads, then resolved by SDS-polyacrylamide gel electrophoresis and analyzed by immunoblotting, as described above.

### GST pulldown assay

GST and GST-E7 were expressed in BL21DE *Escherichia coli* cells and purified with standard procedures. CaSki and H358/Tet-On/p14^ARF^ cells lysates were incubated with either GST or GST-E7 glutathione-sepharose (Sigma) for 30 min at 4°C. UBF1, p14^ARF^, and HPV16 E7 were detected by western blot as described above.

### RT-PCR

Total RNA was extracted using RNeasy mini kit (Qiagen) according to the instuctions of the manufacturer and treated with DNAse (Ambion). RNA (1 µg) was reverse-transcribed using random hexamers (Transcriptor High Fidelity cDNA synthesis kit, Roche). The resulting cDNA (200 ng) was subjected to real-time RT-PCR using TaqMan probes with Abiprism7500 (Applied Biosystems). Primers and probes sequences were as follows: *E7 5′-CGGACAGAGCCCAATACAAT-3′* (forward), *5′-ACGTGTGTGCTTTGTACGCAC-3′* (reverse), *5′-TGTTGCAAGTGTGACTCTACGCTTCG-3′* (probe), *GAPDH 5′-GAAGGTGAAGGTCGGAGT-3′* (forward), *5′-GAAGATGGTGATGGGATTTC-3′* (reverse), *5′-CAAGCTTCCCGTTCTCAGCC-3′* (probe). The relative quantities of E7 mRNA were calculated using the ΔΔCt method with *GAPDH* as the calibrator gene. Fold changes are relative to level of E7 mRNA in cells expressing E7 alone (set up to 1). No-RT controls were included to check the absence of contaminating DNA.

### Immunofluorescence and confocal imaging

MCF7 and U2OS cells were seeded in 24-well plates containing coverslips (5×104 cells/well). One day later, cells were transfected with 200 ng of pCMV16 E7-Flag/HA or pCMV16E7C24G-Flag/HA and/or 200 ng of pcDNA3.1-p14ARF plus Fugene (FuGENE HD Transfection Reagent, Roche). Twenty-four hours later the cells were fixed with 3% paraformaldehyde and permeabilized with 0.1% Triton X-100 in PBS. Primary antibodies, diluted 1/50 in PBS for p14ARF (C18, Santa Cruz Biotechnology) and 1/10 for E7-HA (anti-HA F7, Santa Cruz Biotechnology), were incubated for 90 min at 37°C, followed by extensive washing in PBS and incubation for 30 min at 37°C with secondary antibodies diluted 1/500 (alexa fluor 488 goat anti-rabbit antibody and alexa fluor 568 chicken anti-mouse antibody, Molecular Probes, Invitrogen Ltd). Confocal microscopy was done at the UMR-CNRS 6187 facility. Samples were examined with a spectral confocal FV-1000 station installed on an inverted IX-81 microscope (Olympus, Tokyo, Japan) equipped with an Olympus UplanSapo ×60, 1.2 NA, water objective. Multiple fluorescence signals were acquired sequentially to avoid cross-talk between image channels. Fluorophores were excited with the 488-nm line of an argon laser (for alexa fluor 488), the 543 nm line of an HeNe laser (for alexa fluor 568) and the 633 line of an HeNe laser (for topro-3). Emitted fluorescence was detected through spectral detection channels between 500–530 nm and 550–625 nm, for green and red fluorescence, respectively, and through a 650-nm long-pass filter for far red fluorescence.

### Statistical analysis

The nonparametric Mann-Whitney and the two-tailed T tests were used for statistical analysis. Results are report as mean ± Standard Deviation (SD). *p* values of 0.05 or less were considered significant. All experiments were done at least three times.

## Supporting Information

Figure S1 **HPV16 E7C24G expression stimulates UBF1 phosphorylation.** (A), Western blot analysis of H358 WT cells transiently transfected with the pcDNA3.1 (Control), pJ4Ω16E7 (E7), pCDNA-E7C24G (E7C24G) or pcDNA3.1-p14ARF (p14ARF) expression vector, or both and probed with antibodies to UBF, phosphorylated UBF (P-UBF Ser 388), p14ARF and actin (as a loading control). (B), Western blot analysis of H358 Cl19 cells cultured with (+Dox) or without (-Dox) 1 mg/ml doxycyclin, transfected with pcDNA-E7C24G (E7C24G), and probed with the indicated antibodies. Quantification of western blots was done by measuring the relative intensity of the bands compared to internal controls (Actin), the values given are in arbitrary units. Expression of E7 was monitored by quantitative real time RT-PCR. Western blot and qRT-PCR are representative of three experiments.
(TIF)
