# Supplementary figures and images for: Human Papillomavirus 16 Oncoprotein E7 Stimulates UBF1-Mediated rDNA Gene Transcription, Inhibiting a p53-Independent Activity of p14ARF
Source: PLoS One. 2014 May 5;9(5):e96136. doi: 10.1371/journal.pone.0096136 (PMC4010441; doi:10.1371/journal.pone.0096136)

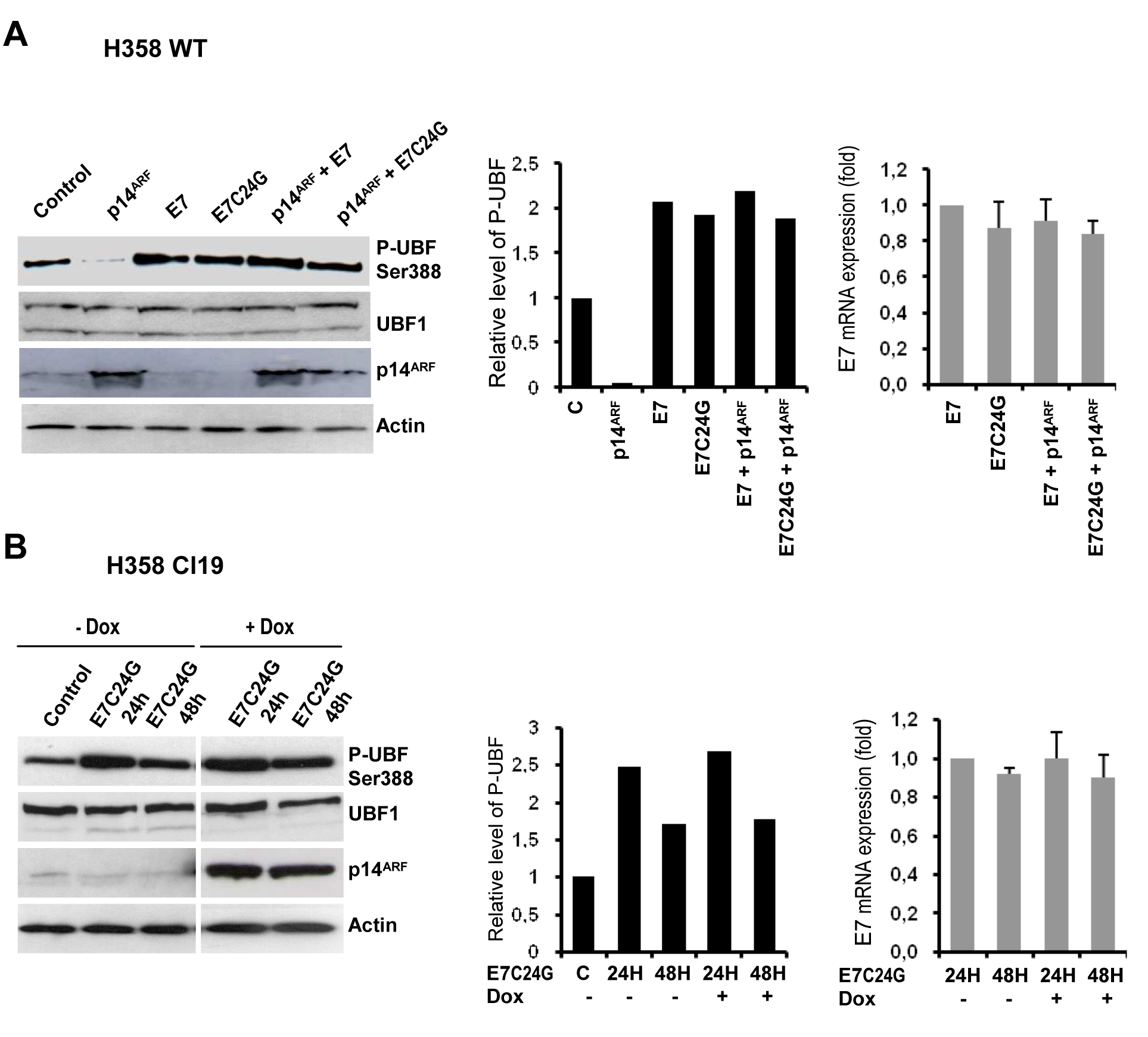

Supplement: Figure S1 — HPV16 E7C24G expression stimulates UBF1 phosphorylation. (A), Western blot analysis of H358 WT cells transiently transfected with the pcDNA3.1 (Control), pJ4Ω16E7 (E7), pCDNA-E7C24G (E7C24G) or pcDNA3.1-p14ARF (p14ARF) expression vector, or both and probed with antibodies to UBF, phosphorylated UBF (P-UBF Ser 388), p14ARF and actin (as a loading control). (B), Western blot analysis of H358 Cl19 cells cultured with (+Dox) or without (-Dox) 1 mg/ml doxycyclin, transfected with pcDNA-E7C24G (E7C24G), and probed with the indicated antibodies. Quantification of western blots was done by measuring the relative intensity of the bands compared to internal controls (Actin), the values given are in arbitrary units. Expression of E7 was monitored by quantitative real time RT-PCR. Western blot and qRT-PCR are representative of three experiments. (TIF) [file pone.0096136.s001.tif]
